# A randomized, phase 2 study comparing pemetrexed plus best supportive care versus best supportive care as maintenance therapy after first-line treatment with pemetrexed and cisplatin for advanced, non-squamous, non-small cell lung cancer

**DOI:** 10.1186/1471-2407-12-423

**Published:** 2012-09-24

**Authors:** Nabil Mubarak, Rabab Gaafar, Samir Shehata, Tarek Hashem, Dani Abigeres, Hamdy A Azim, Gamal El-Husseiny, Hamed Al-Husaini, Zhixin Liu

**Affiliations:** 1 Medical Department, Eli Lilly and Company, Middle East and North Africa, Cairo, Egypt; 2 Medical Oncology Department, National Cancer Institute, Cairo University, Cairo, Egypt; 3 Clinical Oncology Department, Assiut University Cancer Centre, Assiut, Egypt; 4 Clinical Oncology Department, Menoufia University Cancer Centre, Shibin El-Kom, Egypt; 5 Cancer Center Department, Middle East Institute of Health, Beirut, Lebanon; 6 Clinical Oncology Department, Kasr El-Einy Cancer Institute, Cairo University, Cairo, Egypt; 7 Clinical Oncology Department, Faculty of Medicine, Alexandria University, Alexandria, Egypt; 8 Oncology Department, King Faisal Specialist Centre and Research Centre, Riyad, Saudi Arabia; 9 Statistical Sciences Department, Eli Lilly Australia, Sydney, Australia

**Keywords:** Non-squamous, Non-small cell lung cancer, Pemetrexed, Cisplatin, Induction, Maintenance

## Abstract

**Background:**

Maintenance therapy for non-small cell lung cancer (NSCLC) aims to extend disease control after first-line chemotherapy with active and well-tolerated agents. The utility of continuation maintenance therapy requires further research.

**Methods:**

This multicenter, randomized, phase 2 study compared continuation maintenance therapy with pemetrexed (500 mg/m^2^ every 21 days) and best supportive care (BSC) versus BSC alone in patients with advanced, non-squamous NSCLC who had not progressed after 4 cycles of induction chemotherapy with pemetrexed (500 mg/m^2^) and cisplatin (75 mg/m^2^). The primary endpoint was progression-free survival (PFS) from randomization, was analyzed using a Cox model, stratified for the tumor response at the end of induction therapy, at a one-sided alpha of 0.2. Secondary endpoints: response and disease control rates, overall survival (OS), one year survival rates, and treatment-emergent adverse events (TEAEs).

**Results:**

A total of 106 patients commenced induction therapy, of whom 55 patients were randomized to maintenance pemetrexed/BSC (n = 28) or BSC (n = 27). Although the median PFS time for maintenance phase for both arms was 3.2 months, the one-sided p-value for the PFS HR comparison was less than the prespecified limit of 0.2 (HR = 0.76, two-sided 95% confidence interval [CI]: 0.42 to 1.37; one-sided p-value = 0.1815), indicating that PFS was sufficiently long in the pemetrexed/BSC arm to warrant further investigation. Similar PFS results were observed for the overall study period (induction plus maintenance) and when the PFS analysis was adjusted for sex, baseline disease stage, and the ECOG PS prior to randomization. The median OS for the maintenance phase was 12.2 months (95%CI: 5.6 to 20.6) for the pemetrexed/BSC arm and 11.8 months (95% CI: 6.3 to 25.6) for BSC arm. The one-year survival probabilities were similar for both arms for the maintenance phase and the overall study period. Both the induction and continuation maintenance therapies were generally well-tolerated, and similar proportion of patients in each arm experienced at least 1 grade 3/4 TEAE (pemetrexed/BSC, 17.9%; BSC, 18.5%).

**Conclusions:**

Continuation pemetrexed maintenance therapy resulted in promising PFS with an acceptable safety profile in a Middle Eastern population with advanced non-squamous NSCLC and is worthy of further investigation.

**Trial registration:**

NCT00606021

## Background

In patients with advanced non-small cell lung cancer (NSCLC), there is generally a brief period of disease control after response to first-line chemotherapy. Consequently, there is a great need to find more effective and tolerable treatments and strategies to delay progression and improve survival in advanced stage NSCLC. A key consideration for maintenance therapy is to utilize a well-tolerated agent with a relatively low risk of serious, cumulative toxicities. In advanced NSCLC, maintenance therapy is typically initiated after 4-6 cycles of induction chemotherapy with an active doublet and is continued until disease progresses or unacceptable toxicity is reached. Maintenance therapy options include: continuing the induction therapy regimen; continuing the non-platinum or molecularly targeted component of the first-line regimen (‘continuation maintenance’); or introducing a new cytotoxic or molecularly targeted agent after induction therapy (‘switch’ maintenance) [1,2].

Pemetrexed is a multitargeted, antifolate cytotoxic agent that disrupts folate-dependent metabolic processes essential for cell replication through the inhibition of the enzymes thymidylate synthase (TS), dihydrofolate reductase (DHFR), and glycinamide ribonucleotide formyltransferase (GARFT). Pemetrexed received approval of the U.S. Food and Drug Administration (FDA) as initial therapy in combination with cisplatin, as monotherapy after prior chemotherapy, and as maintenance monotherapy in patients with locally advanced or metastatic non-squamous NSCLC whose disease has not progressed after four cycles of platinum-based first-line chemotherapy [3]. Pemetrexed is generally well tolerated; however, its multiple enzyme inhibitions also account for the potentially adverse effects of pemetrexed, including myelosuppression (mainly transient neutropenia), oral mucositis, diarrhea, and rash/desquamation. It has been found that the hematological and non-hematological toxicities of pemetrexed can be reduced through routine vitamin supplementation (folic acid and vitamin B_12_), without loss of efficacy [4]. The efficacy and safety of switch maintenance therapy with pemetrexed has been examined in a previous randomized, double-blind, multinational, phase 3 study [5]. In that study, patients with advanced NSCLC with a good baseline performance status (0 or 1) who did not have disease progression after four cycles of permitted platinum-based chemotherapy (not including pemetrexed) were randomly assigned in a 2:1 ratio to receive switch maintenance therapy with pemetrexed (500 mg/m^2^, Day 1) plus best supportive care (BSC) or placebo plus BSC in 21-day cycles until disease progression. The primary endpoint of PFS and the secondary endpoint of OS were analyzed by intention to treat. The PFS was statistically significantly longer in the pemetrexed/BSC arm than in the placebo/BSC arm (median PFS: 4.3 vs 2.6 months, respectively; hazard ratio [HR] = 0.50, 95% CI: 0.42-0.61, p < 0.0001). Overall survival (OS) was also significantly improved for the pemetrexed/BSC arm compared to the placebo/BSC arm (median OS: 13.4 vs 10.6 months, respectively; HR = 0.79, 95% CI: 0.65-0.95; p = 0.012) [5]. To provide a more comprehensive assessment of the therapeutic benefit of pemetrexed maintenance therapy, quality of life was also examined. In a recently published trial, the results of the quality assessment of these patients were disclosed. Patients were analyzed by the Lung Cancer Symptom Scale. Patients treated with pemetrexed maintenance therapy (500 mg/m2 every 21 days; n = 441) showed a longer time to worsening of pain (HR = 0.76) and hemoptysis (HR = 0.58) compared with patients given placebo (n = 222), without high rates of toxic effects [6].

Consistent with data from other studies of pemetrexed as first- or second-line treatment for advanced NSCLC, prespecified subgroup analyses showed that there were significant treatment-by-histology interactions for both PFS and OS, such that the improvements in PFS and OS were observed mainly in patients with non-squamous histology [5,7].

Due to the favorable results observed for switch maintenance therapy with pemetrexed, we conducted a randomized, phase 2 trial to examine the feasibility of continuation maintenance therapy with pemetrexed in patients with advanced, non-squamous NSCLC. After completing four cycles of first-line induction therapy with pemetrexed and cisplatin, patients without disease progression were randomized to receive maintenance therapy with either pemetrexed and BSC or BSC alone. The primary outcome measure was PFS from randomization. Secondary endpoints included: the overall response rate, the disease control rate, OS, one-year survival rates, and treatment-emergent adverse events.

## Methods

### Study design

This was a multicenter, randomized, open-label, parallel-arm, phase 2 trial conducted at 8 investigative sites in 3 countries: Egypt, Lebanon, and Saudi Arabia. The first patient was entered into the study in January 2008 and the last patient completed the study in December 2010. The study was conducted in accordance with the Declaration of Helsinki and Good Clinical Practice guidelines. The study was approved by the ethics review board at each investigative site and all patients provided their written informed consent. The trial was registered at http://www.clinicaltrials.gov (NCT00606021).

### Patient eligibility

Eligible patients were aged ≥18 years, had an estimated life expectancy of ≥12 weeks, and an Eastern Cooperative Oncology Group performance status (ECOG PS) of 0 or 1. Other eligibility criteria included: a histologic or cytologic diagnosis of Stage IIIB (with pleural effusion and/or positive supraclavicular nodes) or Stage IV NSCLC (using the sixth edition TNM staging system available at the time the study was conducted) with non-squamous histology that was not amenable to curative therapy; no prior systemic anticancer therapy for lung cancer; a calculated creatinine clearance (CrCl) ≥45 mL/min based on the standard Cockcroft and Gault formula and a serum creatinine <1.5 x ULN; an adequate bone marrow reserve and liver function; and at least one unidimensionally measurable lesion according to the Response Evaluation Criteria in Solid Tumors (RECIST; version 1.0)[8]. Prior surgery and radiotherapy (limited to <25% of the bone marrow) were allowed if patients had recovered at least 4 weeks before the initiation of induction therapy.

Exclusion criteria included: any serious concomitant systemic disorder; brain metastasis; clinically significant third-space fluid collections; significant weight loss (>10%) during the 6 weeks before study entry; pregnancy or breast-feeding; inability to interrupt aspirin or other non-steroidal anti-inflammatory agents for a 5-day period (or 8-day period for long-acting agents such as piroxicam); inability or unwillingness to take folic acid, dexamethasone (or equivalent) or vitamin B_12_ supplementation.

### Study treatment

After completing the screening assessments, eligible patients commenced first-line, induction chemotherapy with pemetrexed and cisplatin. Pemetrexed (500 mg/m^2^) and cisplatin (75 mg/m^2^) were given intravenously on Day 1 of each 21-day cycle up to a maximum of 4 cycles or until disease progression, unacceptable toxicity, or another permitted reason for discontinuation. Following induction therapy, patients who still remained in the study without disease progression were randomized in a 1:1 ratio to receive continuation maintenance therapy with pemetrexed (500 mg/m^2^ every 21 days) and BSC or BSC alone. Randomization was stratified by the best overall response to the induction therapy (complete or partial response [CR or PR] vs stable disease [SD] or unknown [i.e. although progression had not been documented, 1 or more target or nontarget sites had not been assessed]). Maintenance therapy commenced on Day 1 of the fifth cycle and continued until disease progression, unacceptable toxicity, or another permitted reason for discontinuation. The end of the study was set at 12 months after maintenance phase randomization or 30 days after the end of the maintenance treatment.

Investigators were responsible for providing appropriate BSC. Permitted pemetrexed dose modifications and delays due to certain toxicities were defined in the protocol. During the induction and maintenance phases, all pemetrexed-treated patients were required to take prophylactic folic acid and vitamin B_12_ supplementation (as described in the pemetrexed prescribing information [9]) and dexamethasone (4 mg orally twice a day or equivalent the day before, the day of, and the day after each pemetrexed dose). Higher or additional dexamethasone doses were permitted for reasons other than routine rash prophylaxis (e.g. antiemetic prophylaxis). Full supportive care therapies were permitted concomitantly during the study, but no other anticancer therapies were permitted.

### Study assessments

Tumor assessments were performed by investigators at each investigative site, per RECIST (version 1.0) requirements [8], at baseline (no more than 4 weeks before the initiation of induction therapy) and every 6 weeks (±1 week) during study therapy (before the start of every other treatment cycle). Confirmation of response was required within ≥28 days and ≤42 days after the first evidence of response. After study therapy discontinuation, responding or stable patients were required to have follow-up efficacy assessments approximately every 6 weeks (±1 week) until documented disease progression, death, or study closure, whichever occurred first. Patients with documented disease progression were monitored for survival approximately every 3 months until death or study closure, whichever occurred first.

Toxicity was assessed at baseline, before the start of each cycle (during induction phase and maintenance phase in both arms), and approximately 30 days after the study therapy discontinuation. Per study protocol ‘treatment emergent adverse events (TEAEs)’ were defined as any untoward medical occurrence that either occurs or worsens at any time after treatment baseline and which does not necessarily have to have a causal relationship with this treatment. The TEAEs were graded according to the Common Terminology Criteria for Adverse Events (CTCAE), version 3.0. After the 30-day post-discontinuation safety follow-up visit, only serious adverse events (SAEs) considered to be related to study drug or protocol procedures had to be reported and followed-up at least every 30 days until the event had resolved, stabilized, or the patient commenced a new systemic anticancer therapy.

### Statistical analysis

The primary objective of the study was to compare PFS for the maintenance therapy phase for pemetrexed/BSC vs BSC alone. The study was powered to detect a PFS HR of 0.56, which was considered to be the effect size that pemetrexed must have to warrant its further development as continuation maintenance therapy, based on the results of a previous study [10].

The study initially required a total of 44 randomized patients to have 80% power to detect a true treatment effect of 0.56 at a one-sided alpha of 0.2, in which case pemetrexed continuation maintenance therapy would be considered to warrant further investigation. The planned randomization ratio for the study was 2:1 to enable the collection of more data about potential adverse events (AEs), while maintaining sufficient power to detect a between-treatment difference in PFS and reducing the exposure of patients to the potentially inferior BSC maintenance ‘therapy’. However, due to an undetected error in the randomization parameter form, randomization was actually implemented in a 1:1 ratio, which resulted in a slight increase in power from 80% to 83%.

Efficacy analyses, other than response-related analyses, were conducted using the qualified intent-to-treat (Q-ITT) analysis population, which consisted of all randomized patients. Response rates and disease control rates were analyzed using the tumor analyzable (TA) population, which included all patients who received at least one dose of therapy and had measurable or evaluable lesions at baseline. For the Q-ITT and TA populations, patients were analyzed according to the therapy to which they were randomly assigned. Safety analyses were conducted with the safety analysis population, which included all patients who received at least one dose of study therapy. For the safety analyses for the maintenance phase and the overall study therapy period (induction and maintenance phases), patients were analyzed according to the therapy they received in the first cycle of the maintenance phase.

PFS was measured from the date of randomization to the date of progression or death. For patients without documented disease progression who were not known to have died by the time of analysis, PFS was censored at the date of the last visit with adequate assessment. For patients who received subsequent anticancer therapy after study therapy discontinuation before disease progression or death, PFS was censored at the date of last visit with adequate assessment before the initiation of the post-discontinuation anticancer therapy.

Cox proportional hazards model, stratified for response at the end of the induction therapy (CR and PR vs SD), was used to estimate the PFS HR. In addition, for each treatment arm, the Kaplan-Meier estimates of the median PFS and associated 95% CI were calculated. The unadjusted between-arm comparisons of PFS were conducted using a log rank test and a Wilcoxon test (to account for early events). The p-value associated with the Wald Chi-Square test was used for testing whether the HR was equal to unity at a one-sided alpha of 0.2. In addition, PFS was calculated for the overall study period (induction plus maintenance therapy) measured from the first dose of induction therapy to the date of progression or death. The potential confounding effect of differences in certain baseline characteristics (sex, ECOG PS score before randomization, stage of disease at entry) was also investigated by including these prespecified characteristics in the primary model.

OS for the maintenance phase was calculated from randomization to death from any cause. OS for the overall study period was calculated from the first dose of induction therapy to death from any cause. Survival time was censored at the date of last contact for patients who were still alive or lost to follow-up. OS was assessed using the Kaplan-Meier method. Between-arm comparisons were made using the log-rank test at a two-sided significance level of 0.05. The overall response and disease control rates were calculated for the maintenance phase and the overall study period, and between-arm comparisons were performed using the Chi-square test at a two-sided significance level of 0.05. All analyses were conducted using SAS® software version 9.1.3 (SAS Institute Inc., Cary, NC).

## Results

### Patient disposition

**Figure 1:**
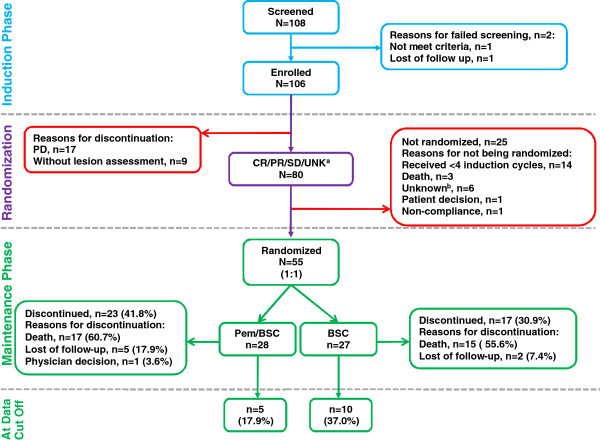
**Patient disposition.**^a^CR/PR/SD/UNK = Complete Response (n = 1, 0.9%)/Partial Response (n = 26, 24.5%)/Stable Disease (n = 49, 46.2%), per RECIST/UNK = unknown (i.e. progression had not been documented, and 1 or more target or nontarget sites had not been assessed [n = 4, 3.8%]) ^b^Unknown = insufficient data Abbreviations: AE = adverse event, BSC = best supportive care, Pem = pemetrexed, PD = progressive disease.

Patient disposition is summarized in Figure 1. A total of 108 patients were screened for the study, of whom 1 did not meet the eligibility criteria and 1 was lost to follow-up. Therefore, 106 patients were enrolled and received at least 1 dose of induction therapy. Following the induction phase, a total of 80 (75.4%) patients had no disease progression documented, although 55 patients of them were considered protocol-qualified and were randomized to maintenance treatment with pemetrexed/BSC (n = 28) or BSC alone (n = 27). All of the patients in the pemetrexed/BSC arm received at least 1 dose of maintenance therapy. The most common reason for premature study discontinuation in both arms was death (pemetrexed/BSC, 60.7%; BSC, 55.6%), which was predominantly due to non-squamous NSCLC. Five (17.9%) patients in the pemetrexed/BSC arm and 10 (37.0%) patients in the BSC arm were alive and progression-free at the end of the study. The main reasons for treatment discontinuation in the pemetrexed/BSC and BSC arms, respectively were: progressive disease (64.3% vs 77.8%), patient decision (17.9% vs 7.4%), and death (14.3% vs 7.4%).

### Baseline patient characteristics

Table 1 presents some key baseline characteristics for the randomized patients, before the start of induction therapy, and the patients’ best tumor response during the induction therapy phase. Baseline characteristics were generally similar between the two treatment arms except that there were slightly higher proportions of patients who were never smokers or had stage IV disease in the pemetrexed/BSC arm (pemetrexed/BSC arm, 42.9% and 67.9% vs BSC arm, 37.0% and 63.0%, respectively), and there was a higher proportion of females in the BSC arm (pemetrexed/BSC arm, 37.0% vs BSC arm, 28.6%). The best overall tumor responses to induction therapy were generally similar between the arms in terms of the numbers of responding and stable patients. The numbers of patients with ECOG PS scores of 0, 1 or 2 at the start of the maintenance phase were generally similar between the two maintenance arms (2 patients in each arm had an ECOG PS of 2; other data not reported here).

**Table 1:** **Demographic and clinical baseline characteristics of all randomized patients with non-squamous NSCLC (maintenance phase**^a^**)**

**Characteristic**	**Pemetrexed/BSC (n = 28)**	**BSC alone (n = 27)**
Median age (range), years	61 (22-75)	59 (48-83)
Males, n (%)	20 (71.4)	17 (63.0)
Race, n (%)		
Caucasian	26 (92.9)	26 (96.3)
African	2 (7.1)	1 (3.7)
ECOG PS, n (%)		
0	8 (28.6)	6 (22.2)
1	20 (71.4)	21 (77.8)
Prior therapy, n (%)		
Radiotherapy	2 (7.1)	1 (3.7)
Curative surgery	0 (0.0)	2 (7.4)
Disease stage^b^, n (%)		
IIIB	9 (32.1)	10 (37.0)
IV	19 (67.9)	17 (63.0)
Histologic subtype, n (%)		
Adenocarcinoma	19 (67.9)	21 (77.8)
Large cell	8 (28.6)	5 (18.5)
Mixed cell	1 (3.6)	1 (3.7)
Smoking status, n (%)		
Current smoker	8 (28.6)	6 (22.2)
Former smoker	8 (28.6)	11 (40.7)
Never smoker	12 (42.9)	10 (37.0)
Best tumor response, n (%)^c^		
Complete response (CR)	0 (0.0)	1 (3.7)
Partial response (PR)	10 (35.7)	11 (40.7)
Stable disease (SD)	17 (60.7)	13 (48.1
Unknown	1 (3.6)	2 (7.4)

Abbreviations: *BSC* = best supportive care; *ECOG PS* = Eastern Cooperative Oncology Group Performance Status.

Note: The sum of percentages for some characteristics do not add up to 100% due to rounding.

^a^Maintenance phase was defined as visit 5 to the visit prior to the post-treatment phase (i.e. the data of progression or the start of a new anti-cancer therapy treatment).

^b^Based on TNM Classification, 6^th^ edition.

^c^The best overall tumor response during the induction therapy phase. This was used as the stratification factor for randomization to one of the maintenance phase arms.

### Study treatment and additional therapy

Of the 106 patients that initiated induction therapy, 79 (74.5%) patients completed 3 cycles of induction therapy and 67 (63.2%) patients completed 4 cycles of induction therapy. During induction therapy, there were very few dose reductions or delays, and the median dose intensities (defined as the actual dose/planned dose) were 94.0% for pemetrexed and 94.6% for cisplatin. During the induction phase, all 106 patients were compliant with the required vitamin B_12_ supplementation, and compliance with the required folic acid supplementation ranged from 89.3-100.0%.

Of the 28 patients in the pemetrexed/BSC maintenance arm, 18 (64.3%) received ≥4 cycles, 7 (25.0%) received ≥6 cycles, 4 (14.3%) received ≥12 cycles, 3 (10.7%) received 14 cycles, and 1 (3.6%) received 15 cycles. During the maintenance phase, the median number of cycles of pemetrexed given was 4.0 and the median dose intensity of pemetrexed was 95.3% (range: 75%-102%). There were no pemetrexed dose reductions required during the maintenance phase, but 3 (10.7%) patients required a pemetrexed dose delay for 1 cycle and 1 (3.6%) patient had 2 delayed cycles. During the maintenance phase, compliance was 85.7% for vitamin B_12_ supplementation and an average of 78.7% for folic acid supplementation. After the discontinuation of study therapy, a similar proportion of patients in each arm received at least one type of additional anticancer therapy (pemetrexed/BSC, 46.4%; BSC, 44.4%). The additional therapies were: chemotherapy (39.3% vs 37.0%) and/or radiotherapy (14.3% vs 14.8%).

### Progression-free survival and overall survival

**Figure 2:**
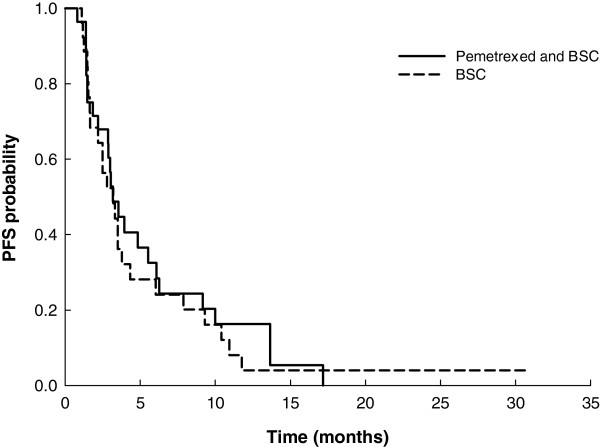
**Unadjusted, unstratified PFS for the maintenance phase (from randomization) for the Q-ITT analysis population.** Pemetrexed + BSC: PFS median time = 3.2 months (95% CI = 2.9-6.1) BSC: PFS median time = 3.2 months (95% CI = 2.2-4.3) one-sided p-value = 0.1815.

**Figure 3:**
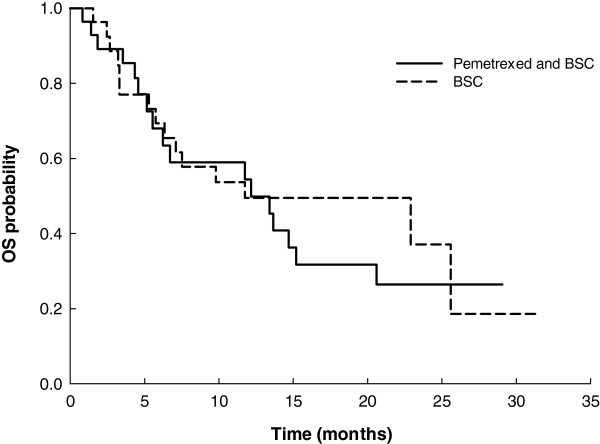
**Unadjusted, unstratified OS for the maintenance phase (from randomization) for the Q-ITT analysis population.** Pemetrexed + BSC: OS median time = 12.2 months (95% CI = 5.6-20.6)**.** BSC: OS median time = 11.8 months (95% CI = 6.3-25.6) one-sided p-value = 0.3619.

The median PFS time for the maintenance phase for both treatment arms was 3.2 months (pemetrexed/BSC arm, 95% CI: 2.9 to 6.1; BSC arm, 95% CI: 2.2 to 4.3). The PFS HR stratified by the best tumor response for induction therapy was 0.76 (two-sided 95% CI: 0.42 to 1.37; one-sided p-value = 0.1815). When the analysis model was also adjusted for the 3 prespecified patient characteristics, PFS remained longer for the pemetrexed/BSC arm than the BSC arm (maintenance phase: HR = 0.65, 95% CI: 0.35 to 1.20, one-sided p-value = 0.0846; overall study period: HR = 0.60, 95% CI: 0.33 to 1.09, one-sided p-value = 0.0461). The median OS time in the pemetrexed/BSC arm was 12.2 months (95% CI: 5.6 to 20.6) and 11.8 months (95% CI: 6.3 to 25.6) in the BSC arm (HR: 1.13, two-sided 95% CI: 0.56 to 2.28). Summarized PFS and OS data are shown in Table 2. The one-year survival probabilities were similar for the two treatment arms for the maintenance phase (pemetrexed/BSC, 54.4%; BSC, 49.5%) and the overall study period (pemetrexed/BSC, 58.6%; BSC, 57.8%). The unadjusted Kaplan-Meier curves for PFS and OS for each arm are shown in Figures 2 and 3, respectively.

**Table 2:** Efficacy measures for patients with non-squamous NSCLC by arm (Pemetrexed + BSC vs BSC)

**Parameter**	**Median PFS or OS time (95% CI)**^a^**, months**		**HR**	**95% CI**^a^	**1-sided p-value**
	**Pemetrexed/BSC (n = 28)**	**BSC (n = 27)**			
***Maintenance Phase***^d^					
PFS	3.2 (2.9 to 6.1)	3.2 (2.2 to 4.3)	0.76^b^	0.42 to 1.37^b^	0.1815^b^
			0.65^c^	0.35 to 1.20^c^	0.08465^c^
OS	12.2 (5.6 to 20.6)	11.8 (6.3 to 25.6)	1.13^b^	0.56 to 2.28^b^	0.36195^b^
			0.95^c^	0.46 to 1.97^c^	0.4497^c^
***Overall Study Period (induction + maintenance)***^e^					
PFS	6.2 (6.0 to 8.3)	6.0 (4.6 to 6.9)	0.71^b^	0.40 to 1.26^b^	0.12325^b^
			0.60^c^	0.33 to 1.09^c^	0.0461^c^
OS	15.4 (8.4 to 23.7)	16.4 (9.1 to 28.5)	1.18^b^	0.59 to 2.38^b^	0.3188^b^
			1.01^c^	0.49 to 2.08^c^	0.48985^c^

Abbreviations: *BSC* = best supportive care; *CI* = confidence interval; *ECOG PS* = Eastern Cooperative Oncology Group performance status; *HR* = hazard ratio; *OS* = overall survival; *PFS* = progression-free survival.

^a^Two-sided 95% confidence interval.

^b^Stratified for the best tumor response during the induction therapy.

^c^Stratified for the best tumor response during the induction therapy and adjusted for sex, baseline disease stage (before induction therapy), and the ECOG PS score before the initiation of maintenance therapy.

^d^Maintenance phase was defined as visit 5 to the visit prior to the post-treatment phase (i.e. the data of progression or the start of a new anti-cancer therapy treatment).

^e^Exploratory data analysis.

### Tumor response

Of the 106 patients who commenced induction therapy, 27 patients had a best overall response of CR (n = 1, 0.9%) or PR (n = 26, 24.5%), 49 (46.2%) patients had a best response of SD, and 4 (3.8%) patients were defined per protocol as unknown. Therefore, the disease control rate for the induction phase was 71.7% (76/106).

For the maintenance phase, there were no cases of a best response of CR or PR, thus the overall response rate was 0.0% for both arms. Sixteen (57.1%) patients in the pemetrexed/BSC arm and 12 (44.4%) patients in the BSC arm had a best response of SD.The disease control rate for the maintenance phase was not significantly different between the pemetrexed/BSC arm and the BSC arm (57.1% vs 44.4%, p = 0.3463). Nine (32.1%) patients in the pemetrexed/BSC arm and 10 (37.0%) patients in the BSC arm showed progressive disease. For the overall study period, the disease control rate was similar for both treatment arms (pemetrexed/BSC, 96.4%; BSC, 96.3%; p = 0.9791).

### Safety

During the induction phase, 73 (68.9%) patients experienced at least 1 treatment-emergent adverse event (TEAE) and 65 (61.3%) patients experienced at least 1 drug-related TEAE. Approximately one-third (31.1%) of the patients experienced at least 1 grade 3/4 TEAE and 12.3% of the patients experienced at least 1 SAE. Twelve (11.3%) patients died during induction therapy. The most common TEAEs (≥10%) during the induction phase were: vomiting (34.0%), decreased appetite (24.5%), fatigue (23.6%), nausea (14.2%), anemia (12.3%), and neutropenia (10.4%). The observed toxicities were consistent with the known safety profile of the induction therapy regimen.

During the maintenance phase, 12 (42.9%) patients in the pemetrexed/BSC arm and 9 (33.3%) patients in the BSC arm experienced at least 1 TEAE. Five patients in each arm experienced at least 1 grade 3/4 TEAE (pemetrexed/BSC, 17.9%; BSC, 18.5%) and 1 patient in each arm experienced at least 1 SAE (pemetrexed/BSC, 3.6%; BSC, 3.7%). There were in total 5 patients (9.1%) with at least 1 grade 3/4 TEAE that were considered possibly drug-related, including 4 patients in pemetrexed/BSC arm (neutropenia, n = 2, 7.1%; anemia, n = 1, 3.6%; hypokalemia, n = 1, 3.6%) and 1 patient in BSC arm (anemia, n = 1, 3.7%). None of the SAEs were drug-related. The most common (≥5% in at least 1 arm) TEAEs during the maintenance phase are summarized in Table 3. The proportions of patients that experienced TEAEs during the maintenance phase were relatively low, and the observed toxicities for the pemetrexed/BSC arm were mostly mild to moderate (grade 1 or 2) and not unexpected for pemetrexed monotherapy. The occurrence of grade 3/4 TEAEs was relatively low in both arms and there were no significant between-arm differences in the proportion of patients experiencing any grade 3/4 TEAE. Three (10.7%) patients required red blood cell transfusions and 1 (3.6%) patient was hospitalized due to NSCLC. Four (14.3%) patients in the pemetrexed/BSC arm and 2 (7.4%) patients in the BSC arm died during the maintenance phase. In the pemetrexed/BSC arm, 3 (10.7%) deaths were due to non-squamous NSCLC and 1 (3.6%) death was due to an AE not related to the study drug (sudden death). In the BSC arm, 1 (3.7%) death was attributed to non-squamous NSCLC and 1 (3.7%) death was due to an AE (cardiac arrest).

**Table 3:** The most common treatment-emergent adverse events during the maintenance phase (≥5.0%)

**TEAE, n (%)**	**Pemetrexed/BSC (n = 28)**		**BSC (n = 27)**	
	**All Grades**	**Grade 3/4**	**All Grades**	**Grade 3/4**
Dyspnea	2 (7.1)	0 (0.0)	4 (14.8)	1 (3.7)
Anemia	3 (10.7)	2 (7.1)^a^	1 (3.7)	1 (3.7)^b^
Chest/thorax pain	3 (10.7)	0 (0.0)	3 (11.1)	0 (0.0)
Neutropenia	2 (7.1)	2 (7.1)^b^	0 (0.0)	0 (0.0)
Abnormal ALT	2 (7.1)	0 (0.0)	1 (3.7)	1 (3.7)
Fatigue	1 (3.6)	0 (0.0)	2 (7.4)	1 (3.7)
Nausea	2 (7.1)	0 (0.0)	0 (0.0)	0 (0.0)
Vomiting	2 (7.1)	0 (0.0)	0 (0.0)	0 (0.0)

Abbreviations: *ALT* = alanine aminotransferase; *BSC* = best supportive care; *TEAE* = treatment-emergent adverse event.

^a^1 (3.6%) patient experienced grade 3 or 4 anemia that was considered by the investigator to be possibly study drug-related.

^b^Considered to be possibly drug-related.

For the overall study period, 75/106 (70.8%) patients who received at least 1 dose of study drug experienced at least 1 TEAE, including 20 (71.4%) patients in the pemetrexed/BSC arm and 18 (66.7%) patients in the BSC arm. The occurrence of drug-related grade 3/4 TEAEs was generally low. Eight (28.6%) patients in the pemetrexed/BSC arm, 12 (44.4%) patients in the BSC arm, and 38/106 (35.8%) patients from the entire study population experienced at least 1 grade 3/4 TEAE during the overall study period. As shown in Table 4, the most common TEAEs (≥15%) in the entire treated patient population (n = 106) for the overall study period were: vomiting (35.8%), fatigue (25.5%), decreased appetite (24.5%), and anemia (16.0%), and the most common grade 3/4 drug-related TEAEs in the entire treated patient population for the entire study period were: neutropenia (8.5%), anemia (5.7%), vomiting (1.9%), and fatigue (1.9%).

**Table 4:** The most common treatment-emergent adverse events for the overall study period (≥10.0% )

**TEAE, n (%)**	**All Treated Patients (n = 106)**^**a**^		**Randomized Patients (n = 55)**^**b**^			
			**Pemetrexed/BSC (n = 28)**		**BSC (n = 27)**	
	**All Grades**	**Grade 3/4**	**All Grades**	**Grade 3/4**	**All Grades**	**Grade 3/4**
Vomiting	38 (35.8)	2 (1.9)^c^	11 (39.3)	0 (0.0)	8 (29.6)	1 (3.7)^c^
Fatigue	27 (25.5)	4 (3.8)^d^	5 (17.9)	1 (3.6)^c^	8 (29.6)	1 (3.7)
Decreased appetite	26 (24.5)	2 (1.9)^e^	8 (28.6)	0 (0.0)	5 (18.5)	0 (0.0)
Anemia	17 (16.0)	8 (7.5)^f^	6 (21.4)	2 (7.1)^f^	6 (22.2)	3 (11.1)^c^
Cough	15 (14.2)	1 (0.9)	3 (10.7)	0 (0.0)	4 (14.8)	0 (0.0)
Dyspnea	12 (11.3)	2 (1.9)	3 (10.7)	0 (0.0)	5 (18.5)	1 (3.7)
Nausea	15 (14.2)	0 (0.0)	6 (21.4)	0 (0.0)	4 (14.8)	0 (0.0)
Chest pain	12 (11.3)	0 (0.0)	6 (21.4)	0 (0.0)	3 (11.1)	0 (0.0)
Neutropenia	12 (11.3)	9 (8.5)^c^	4 (14.3)	3 (10.7)^c^	5 (18.5)	3 (11.1)^c^

Abbreviations: *BSC* = best supportive care; *TEAE* = treatment-emergent adverse event.

^a^The most common TEAEs for the overall study period in the group of patients who were enrolled into the induction phase and who received at least 1 dose of study therapy. This includes the patients who were randomized into the maintenance phase (n = 55) and the patients who were not randomized (n = 51).

^b^The most common TEAEs during induction and maintenance therapy in the patients randomized to the maintenance phase arms.

^c^Considered to be study drug-related.

^d^Two (1.9%) patients experienced grade 3 or 4 drug-related fatigue.

^e^One (0.9%) patient experienced grade 3 or 4 drug-related decreased appetite.

^f^Six (5.7%) patients in the all treated patient population and 1 (3.6%) randomized patient in the pemetrexed/BSC arm experienced drug-related grade 3 or 4 anemia.

There were a total of 17 (60.7%) deaths in the pemetrexed/BSC arm and 15 (55.6%) deaths in the BSC arm for the overall study period, mostly due to non-squamous NSCLC (pemetrexed/BSC, n = 14 [50.0%]; BSC, n = 12 [44.4%]). One death in each arm was due to an AE not study-drug related and no death was reported due to an AE related to study drug. Overall, 5 (17.9%) patients in the pemetrexed/BSC arm and 3 (11.1%) patients in the BSC arm required red blood cell transfusions. During the overall study period, 1 (3.6%) patient in the pemetrexed/BSC arm was hospitalized due to non-squamous NSCLC and 1 (3.7%) patient in the BSC arm was hospitalized due to an AE.

## Discussion

This randomized phase 2 study met its primary endpoint of improved PFS for the maintenance phase, based on the Cox regression model stratified by the best overall response to induction therapy (HR = 0.76, one-sided p-value <0.2), indicating that continuation maintenance therapy with pemetrexed following first-line treatment with pemetrexed and cisplatin was sufficiently beneficial to warrant further investigation. The median PFS times for the maintenance phase did not differ between the two treatment arms. Although clinical researchers may be more familiar with median survival in comparing two treatment groups, this single median point comparison could be insufficient. In contrast, the HR estimate based on Cox regression model compares the whole range of survival times across the two groups; hence a more effective measure of survival difference, given the proportional hazard assumption is met. HR has been commonly used to present the primary result of survival data in oncology trials. Disease control rates, OS and one-year survival probabilities for the maintenance phase were similar for both arms. The exploratory examinations of OS for the overall study period were also similar for both arms. In addition, both the induction therapy and maintenance pemetrexed therapy were generally well-tolerated and there were relatively few grade 3/4 TEAEs observed in either study phase.

In the current study, the median PFS and OS times for the maintenance phase for the pemetrexed/BSC arm were 3.2 and 12.2 months, respectively, which are substantially shorter than the median PFS and OS times of 4.5 months and 15.5 months observed in the previous phase 3 trial in patients with advanced, non-squamous NSCLC following switch maintenance therapy with pemetrexed [5]. It could be possible this Arab patient population may have less access to supportive care than patients from more affluent, industrialized countries, and that would favor a relatively poorer prognosis, although these statistical variations could also be explained by the smaller size of the population of the current study.

A primary consideration in designing a phase 2 clinical study is to minimize the chance that a truly active agent or regimen is erroneously rejected by keeping the probability of type II error (false-negative) low [11]. We attempted to do this by using a higher type I error rate (alpha = 0.2) than would typically be used in a larger phase 3 trial (alpha = 0.05). In addition, stratified PFS was prospectively determined as the primary endpoint of the trial to minimize potential confounding from differences in response to induction therapy. This choice appears to be justified given that a higher proportion of patients in the BSC arm responded to the induction therapy compared to the pemetrexed/BSC arm (40.7% vs 25.0%). Therefore, it is possible that more patients in the BSC arm may have had a better general prognosis.

Study design limitations include the small sample size and the randomization error, which resulted in patients being randomized to the pemetrexed/BSC and BSC arms in a 1:1 ratio rather than a 2:1 ratio. Consequently, fewer patients than originally planned were randomized to the pemetrexed/BSC arm, although this did not preclude evaluation of the primary study hypothesis. These limitations may partly account for the lack of significant between-arm differences observed for the efficacy outcomes. Another potential limitation that could be worth mentioning is the absence of a placebo arm. The investigator-assessed response and disease progression are normally not precisely measured as OS is (with an exact date of death), and therefore may be subject to assessment bias, particularly in open-label studies such as this one. Obviously, we cannot rule out this possibility. However, as this is a phase 2 proof of concept study rather than a confirmatory phase 3 trial, an independent review panel was not used to validate the investigator assessments. The administration of placebos in oncology studies was reviewed by Chvetzoff and Tannock [12], it was shown to improve symptom control (such as pain and appetite) but did not lead to tumor response. Hence, given that our primary and secondary endpoints were PFS, OS and tumor response rate, we argue that the absence of a placebo would not introduce significant bias to our efficacy outcome measures in this trial.

Recent results of a phase 3 trial provide stronger evidence of the potential benefit of continuation pemetrexed maintenance therapy [13]. The PARAMOUNT study investigated whether pemetrexed continuation maintenance therapy would improve PFS after pemetrexed-cisplatin induction therapy in patients with advanced, non-squamous NSCLC [13]. Randomized patients must not have progressed during the induction therapy and must have had an ECOG PS of 0 or 1 at the end of induction therapy. A total of 539 (57.4%) patients were randomized in a 2:1 ratio to continuation maintenance with pemetrexed/BSC (n = 359) or placebo (normal saline)/BSC (n = 180). Randomization was stratified for baseline disease stage, the best overall response to the induction therapy, and the ECOG PS just prior to randomization [13]. The study had 90% power to show a statistically significant between-arm difference in PFS for the maintenance phase at an alpha of 0.05, assuming that the true unadjusted HR was 0.65. A median of 4 cycles of maintenance therapy was delivered in each arm. PFS from randomization was significantly longer for the pemetrexed/BSC arm than the placebo/BSC arm (median PFS: 4.1 vs 2.8 months, respectively; unadjusted HR = 0.62, 95% CI: 0.49 to 0.79; p = 0.00006) [13]. Similarly, the exploratory analysis of the overall PFS (from induction) showed it was significantly longer for the pemetrexed/BSC arm than the placebo/BSC arm (median PFS: 6.9 vs 5.6 months; HR = 0.59, 95% CI: 0.47 to 0.74, p < 0.00001). The independently reviewed disease control rate for the maintenance phase was 71.8% for the pemetrexed/BCS arm and 59.6% for the placebo/BSC arm (p = 0.009). Patients achieved a median OS of 13.9 months from randomization (16.9 months from start of induction) on the pemetrexed continuation maintenance arm compared to 11.0 months from randomization (14.0 months from start of induction) on the placebo arm [14]. The main differences in adverse events reported in the PARAMOUNT trial between the two arms were higher grade 3/4 toxicity rates for pemetrexed as follows: fatigue (4.2% vs 0.6%, respectively), anemia (4.5% vs 0.6%), and neutropenia (3.6% vs 0%) [14]. Consistent with the safety results for our study, the safety data from the PARAMOUNT study showed that pemetrexed maintenance therapy was generally well-tolerated [5,14]. The finding of an optimal maintenance therapy for patients with locally advanced or metastatic non-squamous NSCLC, who achieved disease control after first-line chemotherapy, is still the subject of study.

## Conclusions

The results of our study showed that continuous pemetrexed maintenance therapy following 4 cycles of pemetrexed-cisplatin induction therapy resulted in promising PFS with an acceptable safety profile in a Middle Eastern population with advanced non-squamous NSCLC, and that it is worthy of further investigation. Data currently published for the benefit of maintenance therapy with pemetrexed also support the results of our study.

## Competing interests

NM and ZL are full-time employees of Eli Lilly and Company, the manufacturer of pemetrexed. All other authors agree in declaring they have no competing interests (RG, SS, TH, DA, HAA, GEH, and HAH).

## Authors’ contributions

All authors were involved in the drafting and revision of the manuscript. In addition, NM was involved in the study design and interpretation of the data. ZL was involved in the statistical analyses and the interpretation of the data. All other authors were clinical investigators in the trial and were involved in patient care and data collection. All authors read and approved the final manuscript.

## Pre-publication history

The pre-publication history for this paper can be accessed here:

http://www.biomedcentral.com/1471-2407/12/423/prepub
